# Hepatitis E virus infection in a patient with alcohol related chronic liver disease: a case report of acute-on-chronic liver failure

**DOI:** 10.1186/s12985-021-01714-w

**Published:** 2021-12-09

**Authors:** Anabella Fantilli, Sarah Daniela López Villa, Alina Zerega, Guadalupe Di Cola, Luis López, Maribel Wassaf Martínez, María Belén Pisano, Viviana Elizabeth Ré

**Affiliations:** 1 Instituto de Virología “Dr. J. M. Vanella”- InViV- CONICET, Facultad de Ciencias Médicas, Universidad Nacional de Córdoba, Enfermera Gordillo Gómez s/n, Ciudad Universitaria, CP 5016 Córdoba, Argentina; 2 Instituto Modelo de Cardiología, Córdoba, Argentina; 3 Sanatorio Allende, Córdoba, Argentina; 4 LACE Laboratorios, Córdoba, Argentina

**Keywords:** HEV, Cirrhosis, Chronic liver disease, Acute-on-chronic hepatitis, Argentina

## Abstract

**Background:**

The hepatitis E virus (HEV) infection has been described as a causing factor for acute-on-chronic-liver-failure (ACLF) in patients with underlying chronic liver disease (CLD), such as chronic hepatitis or cirrhosis, which could end in the failure of one or more organs and high short-term mortality. There are scarce data about the association of HEV in patients with chronic liver disorders in South America.

**Case presentation:**

A 56-year-old hypertensive male with a history of type 2 diabetes was diagnosed with alcohol-related-liver cirrhosis in February 2019. A year later, the patient was admitted to hospital due to fatigue, jaundice and acholia. No evidence of hepatitis A virus, hepatitis B virus, hepatitis C virus, Epstein–Barr virus, herpes zoster virus and cytomegalovirus infections were found. Nevertheless, in February and March, 2020 the patient was positive for HEV-IgM and HEV-IgG, and HEV genotype 3 RNA was detected in sera. Afterwards, he presented grade I hepatic encephalopathy and, therefore, was diagnosed with acute hepatitis E-on-chronic liver disease. The patient reported a recent travel to the Argentine coast, where he consumed seafood. Besides, he reveled to have consumed pork meat and had no history of blood transfusion.

**Conclusion:**

This report describes a unique case of hepatitis E virus infection in a patient with alcohol-related cirrhosis. This is the first report of a patient with HEV-related ACLF in Argentina and it invokes the importance of HEV surveillance and treatment among patients with CLD, such as alcohol-related cirrhosis.

## Background

The hepatitis E virus (HEV) (specie *Orthohepevirus A*, genus *Ortohepevirus*, family *Hepeviridae*) is a non-enveloped virus with a positive sense single stranded RNA genome. It is one of the leading causes of acute viral hepatitis of enteric transmission worldwide [1–3]. It has been classified into 8 genotypes (HEV-1 to HEV-8); genotypes 1 to 4 and 7 have been shown to infect humans [3, 4].

Epidemiological differences between genotypes have been reported, such as worldwide distribution, ways of transmission and clinical manifestations. It is well-known that, from the clinical perspective, HEV infections result in a spectrum of reported manifestations including acute infections, mostly asymptomatic, chronic hepatitis in individuals with immunosuppression and/or chronic liver disease (CLD), mainly associated with genotype HEV-3 and HEV-4, extrahepatic manifestations, fulminant hepatitis in pregnant women, when the infecting genotype is 1, and in those individuals with other underlying liver diseases, such as CLD [5–10].

In South America, countries are mainly non-endemic for HEV, showing moderate HEV circulation. Sporadic acute HEV cases and a few cases of chronic hepatitis E or HEV infection with extra hepatic complications have been informed. Only HEV-1 and HEV-3 have been detected, and HEV-3 is the most frequent genotype found in this region [11].

CLD is a pathology characterized by gradual and constant injury to the liver tissue caused by a wide variety of etiologies [12]. Various factors can shorten or accelerate this process of development. Thus, when acute viral hepatitis occurs in patients with CLD, they may develop an acute-on-chronic liver failure (ACLF) [12]. There is no consensus established about the conceptualization and characterization of this syndrome. The Asian Pacific Association for the Study of Liver (APASL), the European association for the study of liver (EASL) and the American association for the study of liver diseases (AASLD), define ACLF differently [13, 14].

Although there is no universally agreed definition of this entity, both, the West and the East, state that the acute deterioration of liver function in a patient with compensated chronic liver disease, mainly stable liver cirrhosis, is the characteristic feature of ACLF, and they consider HEV as a well-defined precipitating event that may cause ACLF [15]. There is only one previous case of HEV-related ACLF reported in Latin America, specifically in Peru [16].

The present study describes a case of acute hepatic deterioration caused by HEV infection in a patient with pre-existing CLD, specifically alcohol-related cirrhosis. This is the first report of a patient with HEV-related ACLF in Argentina.

## Case presentation

A 56-year-old hypertensive male with a history of type 2 diabetes, diagnosed with alcohol-related-liver cirrhosis in February 2019, who presented variceal upper gastrointestinal bleeding at the beginning of the disease, was admitted to hospital in February 2020. He presented a 1-week history of general malaise that began with fatigue and then jaundice and acholia, without fever or abdominal pain. His treatment before hospitalization was propranolol 80 mg/day, metformin 850 mg/day and sitagliptin 50 mg/day. He denied any alcohol, cigarettes or illicit drug consumption recently and reported a recent travel to the Argentine coast, where he consumed seafood. Besides, he reveled to have consumed pork meat and no history of blood transfusion.

Physical examination demonstrated he was normotensive, afebrile, with mucocutaneous jaundice. Laboratory investigations showed elevated aspartate aminotransferase (AST) of 1347 IU/mL; alanine aminotransferase (ALT) of 1387 IU/mL, total bilirubin of 9 mg/dL, alkaline phosphatase of 181 IU/mL, international normalized ratio (INR) of 1.17, plasma prothrombin activity (PPT) of 68%, platelets of 159 mL/mm3, and total protein of 7.21 g/dL (Table 1). Antinuclear antibody (ANA), antismooth muscle antibody (ASMA) and antimitochondrial antibodies (AMA) were negative. A complete blood count, creatinine and ionogram were in normal parameters. Abdominal magnetic resonance with contrast and cholangioresonance revealed liver that suggested CLD.

**Table 1:** Clinicopathological parameters of the patient throughout different stages of hospitalization

Laboratory parameters	Hospital admission 02/2020	06/03/20	Before 1st transplant 18/05/20	After 1st transplant 26/05/20	Cholestasis event 23/07/2020	Liver resection 28/09/2020	Before 2nd transplant 23/03/21
ALP (UI/mL)	181	141	64	232	451	630	242
AST (UI/mL) *	1347	151	50	565	45	42	17
ALT (UI/mL) *	1387	150	40	1565	73	53	106
INR *	1.17	1.29	1.9	1.09	1.03	1.1	1.3
PPA (%)	68	60	40	92	98	87	63
Creatinine (mg/dL) *	0.84	1.14	1.6	0.9	0.7	0.8	1.7
Blood urea (mg/dL)	27	55	45	38	48	N/A	N/A
Total bilirubin (mg/dL) *	9.18	25.83	11	4.6	8	10	4.3
Na levels (mmol/L) *	135.8	131	135	140	137	134	132
K levels (mmol/L)	4.1	3.67	3.9	4.1	3.8	3.4	4.7
Cl levels (mmol/L)	100.2	89.8	105	102	102	97	100
Red cell count (mill/mm3)	4.39	4.51	3.1	3.16	4.32	3.19	2.68
Hemoglobin (g/dL)	14.4	15	10.5	10.5	14.4	9.9	8.6
Platelets (n°/mm3)	159,000	165,000	113,000	209,000	234,000	196,000	128,000
White cell blood count (n°/mm3)	6590	8820	9350	8680	3620	3510	12,900
Total proteins (g/dL)	7.21	6.8	N/A	5.8	N/A	N/A	N/A
Encephalopathy	NO	YES	YES	NO	NO	NO	YES

N/A, not available; ALP, Alkaline phosphatase; AST, Aspartate aminotransferase; ALT, Alanine transaminase; INR, International normalized ratio; PPA, Plasma prothrombin activity

*Parameters used for the calculation of the MELD score

**Fig. 1:**
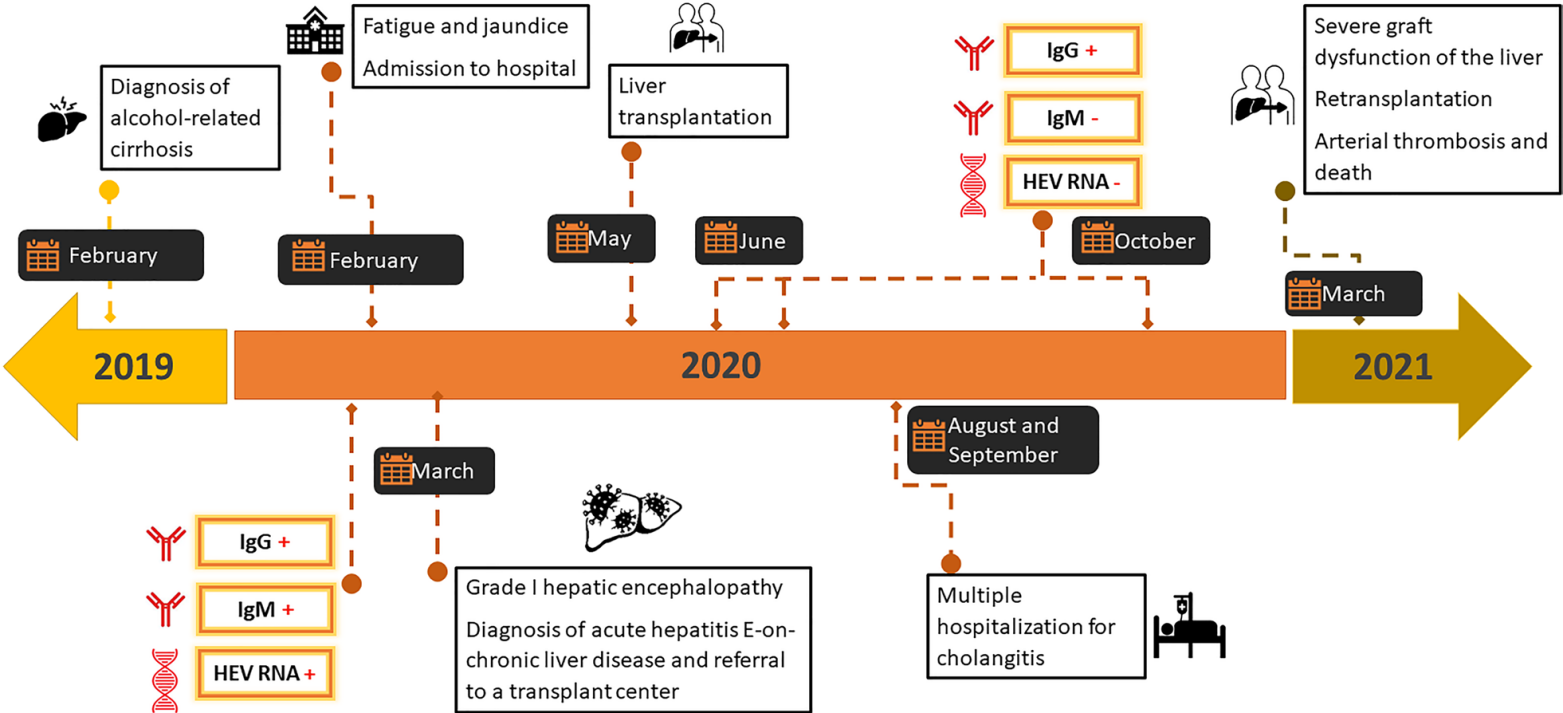
Patient medical history timeline

Serological tests for hepatitis A, B, and C viruses, Epstein–Barr virus, herpes zoster virus, and cytomegalovirus were negative. IgG and IgM anti-HEV antibodies were tested by third generation ELISA assays, using commercial kits (Diapro, Italy), following the manufacturer’s instructions, revealing that this patient was HEV-IgM and HEV-IgG double positive in February and March, 2020. RT-real time PCR for molecular HEV screening was also carried out. Briefly, to obtain cDNA, retrotranscription was performed using random hexamer primers and the enzyme Reverse Transcriptase (ImPromII -Reverse Transcriptase- Promega), followed by genomic detection of HEV by real time PCR (iTaq Universal Probes Supermix- BIO-RAD) [17]. Both samples, obtained in February and March 2020, resulted positive. A nested-PCR, amplifying a 348 bp fragment of the ORF-2 region for HEV 1–4 genotypes, using the enzyme GoTaq (Promega), previously described [18], was carried out, obtaining a positive result. The resulting amplicon was purified using a QIAquick Gel Extraction Kit (QIAGEN, Valencia, CA, USA) and subjected to direct nucleotide sequencing in both directions (Macrogen, Inc. Seoul, Korea): the presence of HEV genotype 3 was determined (Fig. 1).

Following admission, during the first days of March 2020, the patient’s liver enzyme levels showed a downward trend with AST values of 151 IU/mL, ALT of 150 IU/mL with rising hyperbilirubinemia and total bilirubin of 25.83 mg/dL. At this stage, he evolved with signs of grade I hepatic encephalopathy (bradypsychia). Thus, the patient was diagnosed with acute hepatitis E-on-chronic liver disease and was referred to a transplant center for treatment evaluation with a MELD score of 23 points (INR of 1.29, creatinine of 1.14 mg/dL, total bilirubin of 25.83 mg/dL, Na of 131 mg/dL) and MELD-Na score of 27 points. The patient was transplanted on May 19, 2020. The presence of IgM anti-HEV was negative and IgG anti-HEV was positive for successive serum samples in May, June and October, although HEV RNA could not be amplified during these months (Fig. 1), therefore, chronic infection by this agent was ruled out.

Throughout August and September, 2020, the patient had multiple hospitalizations for cholangitis. The following months he manifested a severe graft dysfunction of the liver; therefore, the patient underwent a liver retransplantation in March, 2021. Over the next days, he suffered an arterial thrombotic event and passed away on March 25, 2021.

Table 1 shows the clinicopathological parameters of this patient throughout different stages of hospitalization (during hospital admission, before and after the first and second transplant).

## Discussion and conclusions

Herein, we present an acute-on-chronic liver failure case. Reports of clinically significant HEV cases are low or infrequent in South America, due to a high rate of asymptomatic or underdiagnosed cases in this region. To the best of our knowledge, this is the first published case of acute hepatitis E in a patient with chronic liver disease in Argentina.

In general, and as it can be specifically observed in this case, the causative relation between HEV infection and the development of ACLF is difficult to establish. On the one hand, it has been widely studied that there is a predisposition to acquire HEV among patients with underlying liver diseases, such as cirrhosis [19–22]. Some reports have documented that cirrhosis-associated immune dysfunction can lead to alterations in innate and acquired immunity, both at an intrahepatic and systemic level. This could, in theory, explain why patients with cirrhosis may be prone to contract HEV infection [23, 24]. On the other hand, there are also multiple studies conducted worldwide [12, 20, 25–34] that analyzed how HEV infection contributes to the progression of CLD, showing that HEV infection negatively impacts the survival and prognosis of this group of patients, and has been indicated as a promoting factor for the progression of cirrhosis and hepatocellular carcinoma. Therefore, it could be considered an important trigger of liver decompensation, high morbidity and lethality in patients with underlying liver diseases [12, 20, 25–34]. Accordingly, an increasing number of reports from Asia, an HEV-endemic area, shows strong evidence that suggests that HEV is a major cause of ACLF in patients previously diagnosed with CLD [28–30, 35–41]. In contrast, in Europe, Africa and North America there are still limited studies [12]. In this regard, in South America, two recent publications in Brazil and Argentina have documented a higher HEV seroprevalence in patients with CLD [19, 42], and there is only one previous case of HEV-related ACLF described in a patient from Peru who developed autoimmune hepatitis and severe hepatic decompensation associated to HEV infection [16]. Nevertheless, despite these associations and evidence of decompensation in patients with preexisting liver diseases, whether HEV causes fulminant hepatitis among those with chronic hepatic disorders from our region remains still unknown.

Another remarkable factor to be taken into account is that the etiology of cirrhosis in the present case was alcohol. Previous studies have proposed excessive alcohol consumption to be considered an important risk factor for HEV infection, as it contributes to the clinical expression of the infection and the severity of hepatitis, and it can induce subclinical liver disease (steatosis and/or fibrosis), which increases the susceptibility of the organ to develop viral symptomatic infections [43–45]. A previous study from France found that acute hepatitis E in persons who had not travelled to HEV-endemic regions may cause fulminant hepatitis, especially in those with active alcohol use [46].

Particularly, in the last years, there have been two studies that describe a significant association between alcoholic-related cirrhosis and HEV infection [21, 42]. In one of them, conducted in the same region from Argentina by our research group, it was reported a high HEV seroprevalence in patients with alcoholic-related cirrhosis, an indication that this group may be prone to contract HEV infection [42]. Moreover, Lin et al. [33] reported a recent case of HEV reinfection in a patient with liver cirrhosis who had rapid hepatocellular carcinoma development, which highlights the important role of HEV in the progression in liver cirrhosis cases, and how a functional decompensated liver predisposes patients to HEV infection.

In our study, the patient reveled that he had traveled to the coast and consumed seafood previous to the onset of symptoms, as well as pork meat consumption. Both types of food have been reported to be means of transmission for HEV [47], although we cannot assure that some of these have been the transmission route, which could be considered a limitation of our study. An additional source of infection for this case could have been contact with contaminated matrices of water, since Argentina has reported the presence of viruses in recreational waters [11]. Although it has been shown that HEV can be transmitted by blood transfusions [11], this route was ruled out since the patient did not receive transfusions during the hospitalization period.

The genotype found in the present case was HEV-3, which is especially associated to chronic infection [48, 49]. This result coincides with previous information about circulating genotypes in this region of South America, where only HEV-1 and HEV-3 have been detected. Particularly, HEV-3 is the most frequent genotype detected in Argentina and it was previously isolated from humans, pigs and environmental samples [11]. Although persistent RNA replication was not found for more than 3 months in our patient, it is well documented that cases of chronic HEV have mostly been observed in immunocompromised people, such as transplanted patients, and in those with underlying chronic diseases, who are at major risk of developing chronic HEV infection when the infecting genotype is HEV-3 [50, 51].

In conclusion, herein we report the first patient with hepatitis E-related ACLF in Argentina. In our region, HEV is not considered a causative agent of hepatitis, and in less levels among patients with CLD; accordingly, the evidence in this report contributes to making this agent visible as a cause of liver disease. Additionally, although the natural history of hepatitis E in patients with CLD is not well understood yet and could not be deeply studied in this case, an acute HEV infection, with the possibility of the development of chronicity, might be a reason of morbidity and finally death in groups of patients with these pathologies. This unique case invokes the importance of HEV surveillance and treatment among patients with CLD. Thus, prompt recognition and diagnosis of HEV should be improved to develop more effective and earlier interventions, by increasing awareness and knowledge of basic and clinical aspects of the disease.

## Data Availability

Not applicable.

## Abbreviations

HEV: Hepatitis E virus
ACLF: Acute-on-chronic liver failure
CLD: Chronic liver disease
APASL: Asian Pacific Association for the study of liver
EASL: European association for the study of liver
AASLD: American association for the study of liver diseases
AST: Aspartate aminotransferase
ALT: Alanine aminotransferase
ALP: Alkaline phosphatase
INR: International normalized ratio
PPA: Plasma prothrombin activity
ANA: Antinuclear antibody
ASMA: Antismooth muscle antibody
AMA: Antimitochondrial antibodies
